# Effects of Clopidogrel on Mortality, Cardiovascular and Bleeding Outcomes in Patients with Chronic Kidney Disease - Data from Taiwan Acute Coronary Syndrome Full Spectrum Registry

**DOI:** 10.1371/journal.pone.0071917

**Published:** 2013-08-28

**Authors:** Tsung-Hsien Lin, Wen-Ter Lai, Ho-Tsung Hsin, Ai-Hsien Li, Chun-Li Wang, Chi-Tai Kuo, Juey-Jen Hwang, Fu-Tien Chiang, Shu-Chen Chang, on behalf of ACS Full Spectrum Registry Investigators

**Affiliations:** 1 Division of Cardiology, Department of Internal Medicine, Kaohsiung Medical University Hospital, Kaohsiung, Taiwan; 2 Faculty of Medicine, College of Medicine, Kaohsiung Medical University, Kaohsiung, Taiwan; 3 Division of Cardiology, Cardiovascular Center, Far Eastern Memorial Hospital, Taipei, Taiwan; 4 College of Medicine, Chang Gung University, Taoyuan, Taiwan; 5 Cardiovascular Division, Department of Internal Medicine, Linkou Chang Gung Memorial Hospital, Taoyuan, Taiwan; 6 Division of Cardiology, Department of Internal Medicine, National Taiwan University Hospital, Taipei, Taiwan; 7 Division of Biostatistics, Institute of Public Health, National Yang-Ming University, Taipei, Taiwan; S.G.Battista Hospital, Italy

## Abstract

**Background:**

The efficacy of clopidogrel is inconclusive in the chronic kidney disease (CKD) population with acute coronary syndrome (ACS). Furthermore, CKD patients are prone to bleeding with antiplatelet therapy. We investigated the efficacy and safety of clopidogrel in patients with ACS and CKD.

**Methods:**

In a Taiwan national-wide registry, 2819 ACS patients were enrolled. CKD is defined as an estimated glomerular filtration rate of less than 60 ml/min per 1.73 m^2^. The primary endpoints are the combined outcomes of death, non-fatal myocardial infarction and stroke at 12 months.

**Results:**

Overall 949 (33.7%) patients had CKD and 2660 (94.36%) patients received clopidogrel treatment. CKD is associated with increased risk of the primary endpoint at 12 months (HR 2.39, 95% CI 1.82 to 3.15, p<0.01). Clopidogrel use is associated with reduced risk of the primary endpoint at 12 months (HR 0.42, 95% CI: 0.29–0.60, p<0.01). Cox regression analysis showed that clopidogrel reduced death and primary endpoints for CKD population (HR 0.35, 95% CI: 0.21–0.61 and HR 0.48, 95% CI: 0.30–0.77, respectively, both p<0.01). Patients with clopidogrel(−)/CKD(−), clopidogrel(+)/CKD(+) and clopidogrel(−)/CKD(+) have 2.4, 3.0 and 10.4 fold risk to have primary endpoints compared with those receiving clopidogrel treatment without CKD (all p<0.01). Clopidogrel treatment was not associated with increased in-hospital Thrombolysis In Myocardial Infarction (TIMI) bleeding in CKD population.

**Conclusion:**

Clopidogrel could decrease mortality and improve cardiovascular outcomes without increasing risk of bleeding in ACS patients with CKD.

## Introduction

Cardiovascular disease (CVD) accounted for approximately one-third of all global deaths [1]. The prevalence of CVD has increased considerably in Asian countries over the past several decades as a result of shifts toward a more “westernized” lifestyle. In Taiwan, the CVD is the second most common cause of mortality since 2010 [2]. Acute coronary syndrome (ACS) is the most severe form of CVD. Because of its major impact on morbidity and mortality, as well as its contribution to annual health care costs, it is of the utmost importance to improve strategies for reducing cardiovascular events (CVE) and preventing complication development.

In ACS aggressive anti-platelet and anti-coagulation therapies have been recently developed and can reduce future CVE but may increase the risk of bleeding. Bleeding events have been associated with mortality in studies of ACS and percutaneous coronary intervention (PCI) [3], [4]. Because lower body weight could be asssociated with bleeding complication in the ACS, weight-adjusted dose of anti-thrombotic agent is recommended in the international ACS guidelines [5], [6]. Compared with the Caucasian, Asian population usually have lower body weight and might possibly suffer from anti-thrombotic and anti-platelet overdose and therefore bleeding complication.

Chronic kidney disease (CKD) is a risk factor for coronary heart disease and bleeding with antithrombotic therapy in patients with ACS [7], [8]. Although clopidogrel decreases cardiovascular events in patients suffering from ACS, its effect is inconclusive in the CKD population. Furthermore, patients with CKD are more prone to bleeding when receiving antiplatelet therapy. Therefore, we sought to determine the efficacy and safety of in-hospital and long-term clopidogrel therapy in patients with ACS and CKD in a prospective cohort in an Asia endemic area of kidney disease [9].

## Methods

### Study Design

The study was a prospective, national, multicenter, non-interventional, observational design. Patients recruitment and definition of ACS had been previously described in detail [10]. Patients’ data, such as baseline characteristics, risk factors, clinical presentation, clinical diagnosis, in-hospital interventions as well as medications prescribed, were collected from admission to discharge. Use of clopidogrel or not was based on patients’ records. Patients were followed up at months 3, 6, 9 and 12 post-discharge and data was collected on medication usage, revascularization strategy as well as clinical events, like death, myocardial infarction (MI), stroke, revascularization and hospitalization. Monitoring for source documentation and accuracy was performed in 5% of all case report forms at each recruiting site. This study was carried out in accordance with the local regulatory guidelines and international guidelines for Good Epidemiological Practice [11]. Ethics committee approval was obtained at all trial sites including China University Medical Hospital, Taoyuan General Hospital, Wan-Fang Hospital, Show Chwan Memorial Hospital, Chia-Yi Christian Hospital, Kuang Tien General Hospital, National Taiwan University Hospital, Cheng Ching Hospital, Sin Lau Hospital The Presbyterian Church of Taiwan, Tainan Municipal Hospital, Mackay Memorial Hospital, E-Da Hospital, Chi-Mei Hospital, Taichung Armed Forces General Hospital, Taipei Tzu Chi General Hospital, Kaohsiung Medical University Chung-Ho Memorial Hospital, Taichung Veterans General Hospital, Pingtung Christian Hospital, Lo-Tung Po-Ai Hospital, Far Eastern Memorial Hospital, National Cheng Kung University Hospital, National Taiwan University Hospital, Yun-lin Branch, Dalin Tzuchi General Hospital, Kee-lung Hospital, Taipei Veterans General Hospital, Cathay General Hospital, Kaohsiung Veterans General Hospital, Taipei Medical University Hospital, Shin Kong Wu Ho-Su Memorial Hospital, Changhua Christian Hospital, National Taiwan University Hospital, Chung Shan Medical University Hospita, Hualien Tzu Chi General Hospital, Mackay Memorial Hospital, Taitung Branch, Linkou Chang Gung Memorial Hospital, Hsin Chu General Hospital, Kaohsiung Chang Gung Memorial Hospital, Tri-Service General Hospital and Cheng-Hsin Hospital. Written informed consent was given by the patients for their information to be stored in the hospital database and used for research.

### Thrombolysis In Myocardial Infarction (TIMI) Bleeding Classification

TIMI bleeding classification includes major and minor bleeding. TIMI major bleeding is defined as patients with intracranial hemorrhage or a ≥5 g/dl decrease in the hemoglobin concentration or a ≥ 15% absolute decrease in the hematocrit. If observed blood loss with ≥ 3 g/dl decrease in the hemoglobin concentration or ≥ 10% decrease in the hematocrit or no observed blood loss with ≥ 4 g/dl decrease in the hemoglobin concentration or ≥12% decrease in the hematocrit, it is defined as TIMI minor bleeding [12].

### Calculation of Kidney Function and Definition of CKD

The estimated glomerular filtration rate (GFR) was calculated using the Modification of Diet in Renal Disease (MDRD) Study equation [GFR = 186.3×(serum creatinine in mg/dl)^−1.154^×(age)^−0.203^×(0.742 if female)] [13]. Chronic kidney disease was defined as a GFR less than 60 ml/min per 1.73 m2. This range corresponds to stage 3 or higher CKD by the National Kidney Foundation’s classification scheme and helps identify individuals with clinically significant CKD [14]. All serum creatinines were measured at admission.

### Outcomes

The primary outcome was the composite CVE of death, non-fatal myocardial infarction and non-fatal stroke at one year. The secondary outcome was TIMI bleeding at discharge. The net clinical in-hospital outcome (NCIO) was defined as composite endpoint of death, non-fatal myocardial infarction, non-fatal stroke and in-hospital TIMI bleeding. We analyzed the whole, CKD and non-CKD populations separately.

### Statistical Analysis

All data were expressed as mean ± standard deviation (SD). For comparability between groups, a chi-square test was used for categorical variables and analysis of variance (ANOVA) was adopted for continuous variables. One-year CVE analysis was performed using Kaplan-Meier survival curves and the log-rank test. Univariate and Cox regression analysis were conducted to calculate odd ratio (OR) and harzard ratio (HR) for in-hospital bleeding (IHB) or CVE. Analyses were conducted as time to first event without double counting of events within analyses involving composite endpoints. The adjusted variables in Cox regression analysis included age, gender, Killip class, hypertension, diabetes mellitus, smoking, family history of cardiovascular disease, prior myocardial infarction, prior heart failure, prior cerebrovascular accident and revascularization, including coronary intervention and bypass grafting, or not during index admission.

Statistical analysis was performed using SAS software version 9.2 (SAS Institute Inc., Cary, NC, USA). All statistical analyses were performed using an level of <0.05 with two-sided testing and this was considered as statistically significant.

## Results

### Clinical Characteristics

A total of 3183 eligible patients were enrolled between October 2008 and January 2010 at 39 medical centers and regional hospitals in Taiwan. Among them, 2819 (88.6% of 3183) subjects with renal parameters and 12 months outcome data were analyzed in this study and 1537 (54.5% of 2819) patients were ST-segment elevation acute coronary syndrome (STE-ACS). The subjects included 2230 men and 589 women (male 79.11%). Mean age was 62.9±13.5 years old.

Overall 949 (33.7% of 2819) patients had CKD. Baseline creatinines were 3.0±2.6 and 1.0±0.2 mg/dl in the CKD (n = 949) and non-CKD (n = 1870) groups. Compared with non-CKD subjects, those with CKD were older, shorter and thinner, had less male sex, higher grade of Killip class, lower diastolic blood pressure, faster heart rate at presentation. They also had more comobidity including hypertension, diabetes, previous coronary artery disease (CAD), stroke and heart failure but lower percentage of smoking and family history of CAD (Table 1).

**Table 1:** Baseline characteristics between those with and without CKD.

Number (%)/Mean (SD)	CKD (N = 949)	Non-CKD (N = 1870)	All (N = 2819)	p value
Sex (male)	679 (71.55%)	1551 (82.94%)	2230 (79.11%)	<0.01 *
Age (year)	69.5 (11.90)	59.5 (13.06)	62.9 (13.54)	<0.01 *
Killip				
Class 1	347 (46.51%)	1059 (68.77%)	1406 (61.50%)	<0.01 *
Class 2	135 (18.10%)	267 (17.34%)	402 (17.59%)	
Class 3	132 (17.69%)	113 (7.34%)	245 (10.72%)	
Class 4	132 (17.69%)	101 (6.56%)	233 (10.19%)	
Blood pressure (mmHg)				
SBP	139.1 (36.73)	139.5 (30.53)	139.3 (32.73)	0.754
DBP	79.0 (22.62)	83.5 (19.72)	82.0 (20.84)	<0.01 *
Heart rate (beat per minute)	86.5 (26.04)	79.8 (19.81)	82.1 (22.32)	<0.01 *
Height (cm)	162.8 (8.18)	164.6 (7.65)	164.0 (7.88)	<0.01 *
Weight (kg)	66.1 (12.47)	69.9 (12.82)	68.6 (12.82)	<0.01 *
Waist circumference	90.1 (10.15)	90.6 (9.30)	90.4 (9.57)	0.495
Serum creatinine (mg/dL)	3.0 (2.63)	1.0 (0.19)	1.6 (1.81)	<0.01 *
Dyslipidemia	376 (39.96%)	717 (38.67%)	1093 (39.11%)	0.511
Hypertension	723 (76.91%)	1053 (56.80%)	1776 (63.56%)	<0.01 *
Diabetes				
Diet treatment only	35 (7.22%)	60 (12.05%)	95 (9.66%)	0.010 *
Medical treatment	499 (52.92%)	513 (27.57%)	1012 (36.09%)	<0.01 *
Smoker				
Current	269 (28.92%)	914 (49.65%)	1183 (42.69%)	<0.01 *
Former	197 (21.18%)	267 (14.50%)	464 (16.74%)	
Never	464 (49.89%)	660 (35.85%)	1124 (40.56%)	
Family history	102 (14.93%)	383 (25.95%)	485 (22.46%)	<0.01 *
Previous CAD	317 (33.40%)	358 (19.14%)	675 (23.94%)	<0.01 *
Previous heart failure	94 (9.91%)	54 (2.89%)	148 (5.25%)	<0.01 *
Previous cerebrovascular accident	134 (14.12%)	118 (6.31%)	252 (8.94%)	<0.01 *
PCI	745 (78.59%)	1645 (88.11%)	2390 (84.90%)	<0.01 *
CABG	40 (4.21%)	56 (2.99%)	96 (3.41%)	0.091
**In-hospital cardiovascular events**				
Death	27 (2.85%)	8 (0.43%)	35 (1.24%)	<0.01 *
Re-infarction	12 (1.26%)	9 (0.48%)	21 (0.74%)	0.022 *
Stroke	4 (0.42%)	6 (0.32%)	10 (0.35%)	0.671
Acute renal failure	47 (4.95%)	6 (0.32%)	53 (1.88%)	<0.01 *
TIMI major/minor bleeding	29 (3.06%)	24 (1.28%)	53 (1.88%)	<0.01 *
Major	9 (31.03%)	8 (33.33%)	17 (32.08%)	0.858
Minor	20 (68.97%)	16 (66.67%)	36 (67.92%)	

CKD, Chronic kidney disease; SBP, Systolic blood pressure; DBP, diastolic blood pressure; CAD, coronary artery disease; TIMI, thrombolysis in myocardial infarction; PCI, percutaneous coronary intervention; CABG, coronary artery bypass grafting.

Overall 2595 (92.05% of 2819) and 2660 (94.36% of 2819) patients received aspirin and clopidogrel treatment, respectively. Compared with subjects without clopidogrel use, those receiving clopidogrel treatment were younger, more male sex, current smoker, taller, higher body weight and percentage of family history of CAD but lower heart rate, serum creatinine, percentage of hypertension, diabetes, prior CAD, stroke and heart failure at presentation. There is no statistical significance among CKD stages between two groups (p = 0.245) (Table 2).

**Table 2:** Baseline characteristics between those with and without clopidogrel.

Number (%)/Mean (SD)	Clopidogrel (+) (N = 2,660)	Clopidogrel (−) (N = 159)	All (N = 2,819)	p value
Sex (male)	2114(79.47%)	116(72.96%)	2230(79.11%)	0.050 *
Age (year)	62.60±13.55	67.48±12.55	62.87±13.54	<0.01 *
Killip				
Class 1	1358(61.62%)	48(58.54%)	1406(61.50%)	
Class 2	388(17.60%)	14(17.07%)	402(17.59%)	
Class 3	233(10.57%)	12(14.63%)	245(10.72%)	
Class 4	225(10.21%)	8(9.76%)	233(10.19%)	
Blood pressure (mmHg)				
SBP	139.09±32.70	143.43±33.09	139.33±32.73	0.114
DBP	81.92±20.87	83.15±20.41	81.99±20.84	0.484
Heart rate (beat per minute)	81.64±22.05	89.88±25.45	82.09±22.32	<0.01 *
Height (cm)	164.12±7.85	161.99±8.12	164.00±7.88	<0.01 *
Weight (kg)	68.78±12.72	65.74±14.12	68.61±12.82	<0.01 *
Waist circumference	90.48±9.50	89.94±10.60	90.44±9.57	0.704
Serum creatinine (mg/dL)	1.62±1.79	2.05±2.11	1.64±1.81	<0.01 *
Dyslipidemia	1021(38.73%)	72(45.28%)	1093(39.11%)	0.100
Hypertension	1653(62.73%)	123(77.36%)	1776(63.56%)	<0.01 *
Diabetes	935(35.35%)	77(48.43%)	1012(36.09%)	<0.01 *
Diet treatment only	91(10.00%)	4(5.48%)	95(9.66%)	0.208
Medical treatment	935 (35.35%)	77 (48.43%)	1012 (36.09%)	<0.01 *
Smoker				
Current	1144(43.68%)	39(25.66%)	1183(42.69%)	<0.01 *
Former	426(16.27%)	38(25.00%)	464(16.74%)	
Never	1049(40.05%)	75(49.34%)	1124(40.56%)	
Family history	468(22.99%)	17(13.82%)	485(22.46%)	0.018 *
Previous CAD	602(22.63%)	73(45.91%)	675(23.94%)	<0.01 *
Previous heart failure	133(5.00%)	15(9.43%)	148(5.25%)	0.015 *
Previous cerebrovascular accident	230(8.65%)	22(13.84%)	252(8.94%)	0.026 *
PCI	2291(86.19%)	99(63.06%)	2390(84.90%)	<0.01 *
CABG	88(3.31%)	8(5.03%)	96(3.41%)	0.245
CKD				0.245
Stage 5	174(20.00%)	20(25.32%)	194(20.44%)	
Stage 4	119(13.68%)	14(17.72%)	133(14.01%)	
Stage 3	577 (66.32%)	45 (56.96%)	622 (65.54%)	
**In-hospital cardiovascular events**				
Death	32(1.20%)	3(1.89%)	35(1.24%)	0.449
Re-infarction	18(0.68%)	3(1.89%)	21(0.74%)	0.085
Stroke	9(0.34%)	1(0.63%)	10(0.35%)	0.549
Acute renal failure	45(1.69%)	8(5.03%)	53(1.88%)	<0.01 *
TIMI Major/Minor Bleeding	46(1.73%)	7(4.40%)	53(1.88%)	0.016 *
Major	13(28.26%)	4(57.14%)	17(32.08%)	0.127
Minor	33(71.74%)	3(42.86%)	36(67.92%)	

SBP, Systolic blood pressure; DBP, diastolic blood pressure; CAD, coronary artery disease; TIMI, thrombolysis in myocardial infarction; PCI, percutaneous coronary intervention; CABG, coronary artery bypass grafting; CKD, chronic kidney disease.

### In-hospital Cardiovascular Outcomes

During admission patients with CKD had more death and re-infarction (2.85% vs 0.43% and 1.26% vs 0.48%, p<0.01 and p = 0.022, respectively) but similar stroke risk (0.42% vs. 0.32%, p = 0.671) compared with non-CKD subjects (Table 1). Furthermore, CKD subjects had higher risk of TIMI bleeding (3.06% vs 1.28%, p<0.01).

At discharge the unadjusted odd ratio (OR) of clopidogrel use for the primary endpoint, TIMI bleeding and NCIO are 0.56 (95% CI : 0.24–1.32, p = 0.183), 0.38 (95% CI: 0.17–0.86, p = 0.020) and 0.43 (95% CI: 0.24–0.78, p<0.01) respectively in the whole population. The benefit of clopidogrel use is statistically significant in the non-CKD population for the TIMI bleeding and NCIO (OR 0.10, 95% CI: 0.02–0.57 and OR 0.20, 95% CI: 0.05–0.80, both p<0.01) but not in the CKD population after adjusting age, gender, Killip class, hypertension, diabetes mellitus, smoking, family history of cardiovascular disease, prior myocardial infarction, heart failure and stroke (Table 3).

**Table 3:** Multivariable-adjusted odd ratios for the association between clopidogrel use and in-hospital cardiovascular events.

Outcome/odds ratio (95% CI)	Unadjusted	Model I	Model II
**Death, Re-MI and Stroke**			
Overall cohort	0.56(0.24–1.32)	0.66(0.28–1.55)	1.11(0.25–5.05)
CKD	1.16(0.35–3.84)	1.24(0.37–4.14)	2.25(0.26–19.58)
Non-CKD	0.28(0.08–0.95)*	0.28(0.08–0.99)*	0.41(0.05–3.62)
**TIMI bleeding (major+minor)**			
Overall cohort	0.38(0.17–0.86)*	0.44(0.19–0.99)*	0.50(0.14–1.82)
CKD	0.55(0.19–1.64)	0.59(0.20–1.74)	1.66(0.19–14.72)
Non-CKD	0.30(0.09–1.04)	0.32(0.09–1.10)	0.10(0.02–0.57)*
**Death, Re-MI, Stroke and TIMI bleeding (Net clinical in-hospital outcome, NCIO)**			
Overall cohort	0.43(0.24–0.78)*	0.50(0.27–0.91)*	0.67(0.24–1.83)
CKD	0.75(0.33–1.70)	0.80(0.35–1.82)	1.77(0.37–8.57)
Non-CKD	0.27(0.11–0.67)*	0.28(0.12–0.70)*	0.20(0.05–0.80)*

Model 1: adjusted for age and sex. Model 2: adjusted for age, gender, Killip class, hypertension, diabetes mellitus, smoking, family history of cardiovascular disease, prior myocardial infarction, prior heart failure, prior cerebrovascular accident.

* , p<0.05.

### Cardiovascular Outcomes at 12 Months

**Figure 1:**
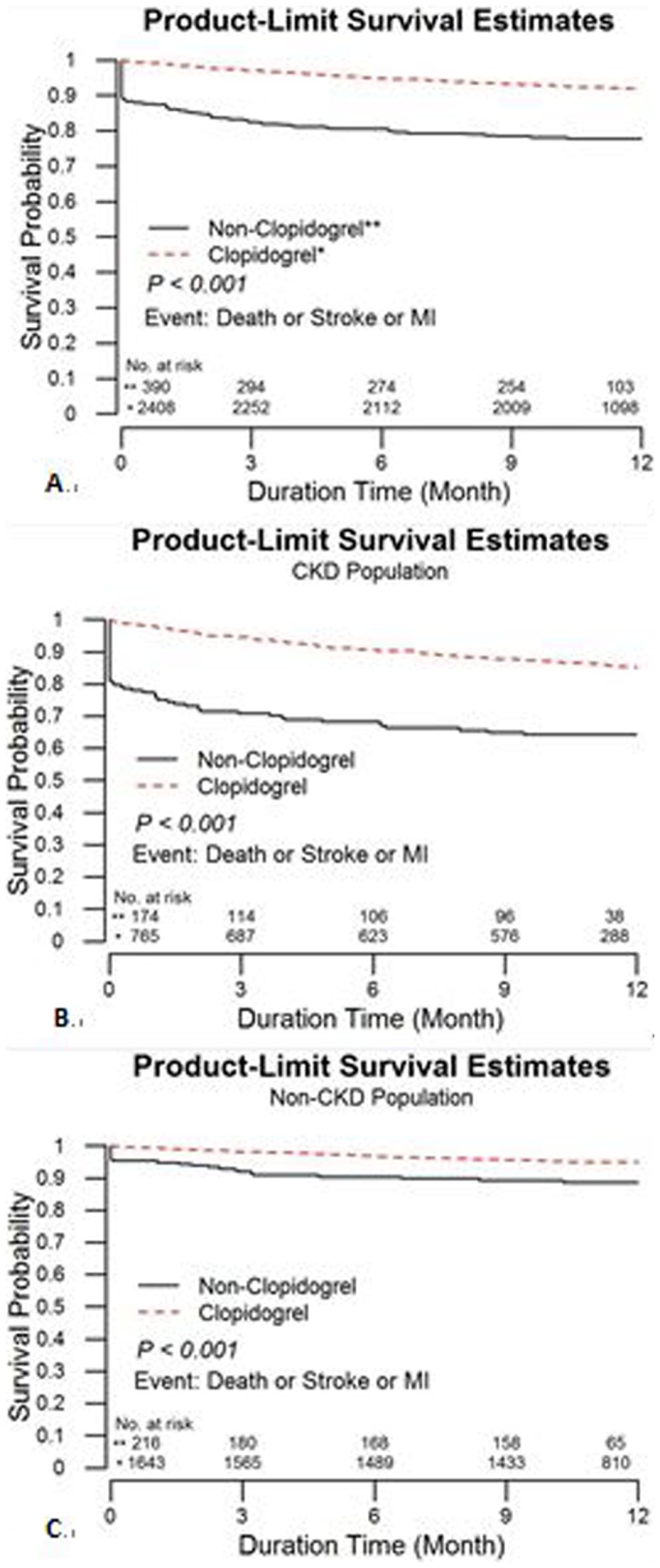
Kaplan–Meier curves of clopidogrel on primary cardiovascular events at 12 months (A) Overall, (B) CKD, (B) non-CKD.

**Figure 2:**
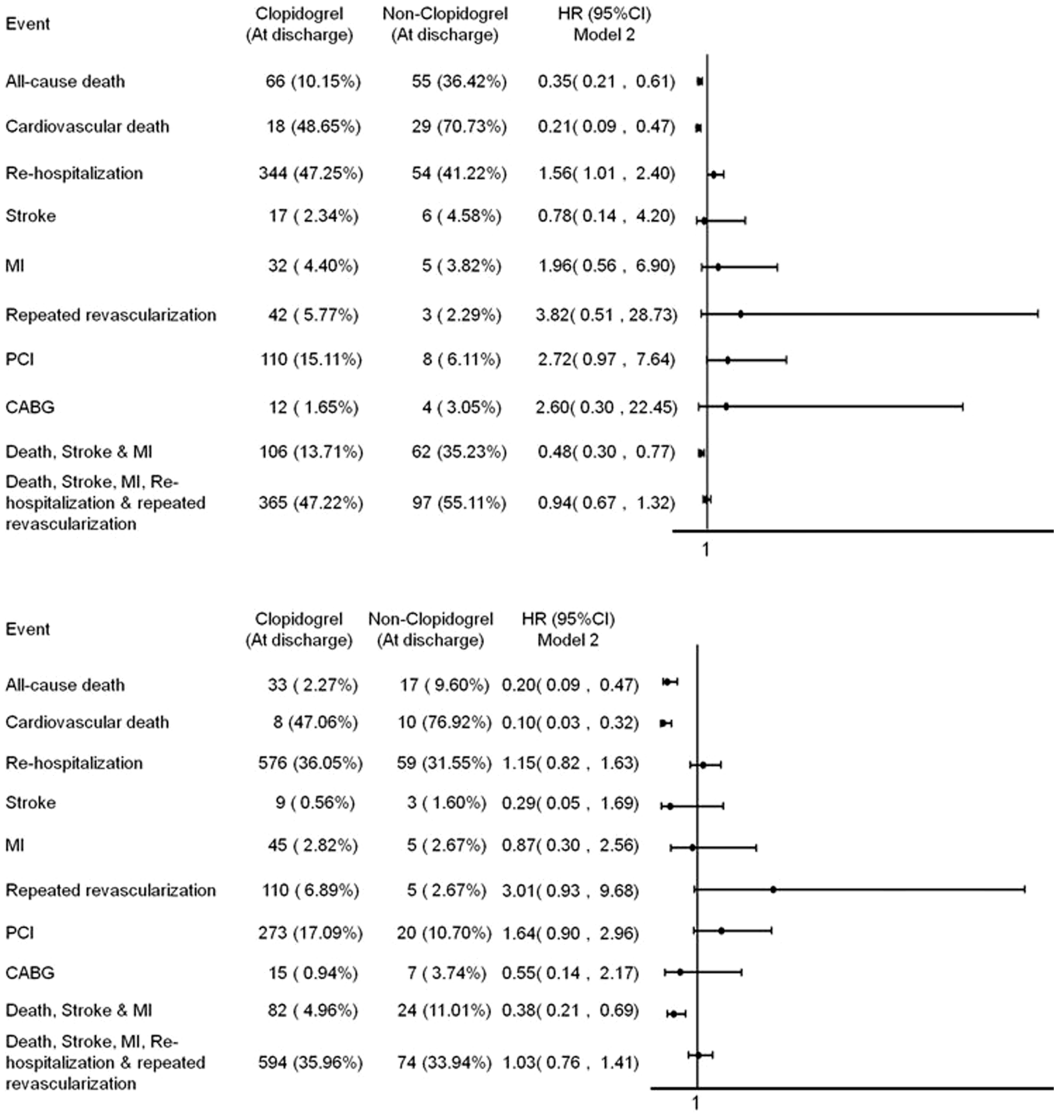
Effect of clopidogrel on cardiovascular events at 12 months (A) CKD, (B) non-CKD. *Adjusted with age, gender, Killip class, hypertension, diabetes mellitus, smoking, family history of cardiovascular disease, prior myocardial infarction, prior heart failure, prior cerebrovascular accident and revascularization including coronary intervention and bypass grafting or not during index admission.

The percentage of aspirin use was not statistically significant between those with and without primary outcome (94.53% and 94.34%, p = 0.900). Kaplan-Meier survival analysis showed that clopidogrel use was associated with lower primary endpoints for the whole, CKD and non-CKD populations at 12 months than the non-clopidogrel use population (all p<0.01) (Figure 1). Cox regression analsysis found the HR of use of clopidogrel in the whole, CKD and non-CKD populations are 0.32 [95% confidence interval (CI) : 0.23–0.46, p<0.01], 0.36 (CI: 0.23–0.56, p<0.01) and 0.35(CI: 0.20–0.62, p<0.01) respectively for the primary endpoints. The association remained statistically significant in the whole, CKD and non-CKD populations [HR 0.42 (95% CI : 0.29–0.60), 0.48 (CI: 0.30–0.77) and 0.38 (CI: 0.21–0.69), respectively, all p<0.01] for the primary endpoints after adjusting age, gender, Killip class, hypertension, diabetes mellitus, smoking, family history of cardiovascular disease, prior myocardial infarction, prior heart failure, prior cerebrovascular accident and revascularization, including coronary intervention and bypass grafting, during index admission. Most importantly death is significantly lower in the patietns receiving clopidogrel treatment in the CKD and non-CKD populations after adjusting above variables (Figure 2).

### Clopidogrel Use and CKD Interaction on Cardiovascular Outcome

CKD is independently associated with a significantly increase of primary endpoint after adjusting age, sex and medication at 12 months (OR 2.17, 95% CI: 1.63 to 2.87, p<0.01). We found an additively detrimental effect on the CVE between no use of clopidogrel and CKD on the occurrence of primary endpoint at 12 months (Table 4). In non-CKD patients, no use of clopidogrel increases the risk of primary endpoint (OR 2.37, 95% CI: 1.47–3.82; p<0.01). When patients receiving clopidogrel treatment, CKD patients had a 3.04-fold risk of primary endpoint (OR 3.04, 95% CI: 2.25–4.12; p<0.01), but CKD patients without clopidogrel treatment had a 10.41-fold risk for primary endpoint (OR 10.41, 95% CI: 7.12–15.24; p<0.01), compared to the non-CKD patients taking clopidogrel.

**Table 4:** Association between clopidogrel use and CKD on primary endopint at 12 months.

Groups N (%)	1 endpoint (+) (N = 274)	1 endpoint (−) (N = 2545)	OR(95% CI)^a^	p value
Clopidogrel use(+)/CKD (−)	82 (4.96%)	1570 (95.04%)	1	–
Clopidogrel use (+)/CKD (+)	106 (13.71%)	667) (86.29%)	3.04 (2.25, 4.12)	<0.01*
Clopidogrel use (−)/CKD (−)	24 (11.01%)	194 (88.99%)	2.37 (1.47, 3.82)	<0.01*
Clopidogrel use (−)/CKD (+)	62 (35.23%)	114 (64.77%)	10.41 (7.12, 15.24)	<0.01*

CKD, chronic kidney bleeding.

## Discussion

There are three major findings in this cohort study. First, patients receiving clopidogrel treatment had lower risk of death at 12 months both in the CKD and non-CKD groups. Second, clopidogrel treatment is not only associated with better cardiovascular outcome in the non-CKD population but also in the CKD population at 12 months follow-up. Third, patients with CKD and no clopidogrel treatment had the worst prognosis during 12 months follow-up. Furthermore, they had additively detrimental effect on the cardiovascular outcome.

### CKD and ACS Outcomes

Taiwan has been recognized as an endemic area of kidney disease with the highest incidence and prevalence rates of ESRD in the world [9]. Because patients with CKD have more comobidity, their treatment strategy in ACS is more complicated. As shown in our study CKD is a poor prognosis factor for those with ACS, possibly because of more extensive and severe atherosclerosis coronary tree with plaque composed of greater necrotic core and less fibrous tissue in the CKD than non-CKD subjects [15], [16], [17]. Furthermore, underuse of reperfusion therapy, fear of contrast-induced nephropathy during coronary procedure, fewer guideline-recommended treatments prescribed and more cormobidities may partly explain why CKD population had poor prognosis in the ACS [18], [19], [20].

### Antiplatelet Effect of Clopidogrel in CKD

The antiplatelet effects of aspirn, clopidogrel and dual combination were reported to have conflict results in the CKD population. Recent studies have shown that CKD is accompanied by a low platelet inhibitory response to clopidogrel administration [21], [22]. Ticagrelor and adjunctive cilostazol were suggested to counteract the ineffective of clopidogrel in the CKD population [23], [24]. However, Kaufman et al. found clopidogrel significantly inhibits ADP-induced platelet aggregation even in subjects receiving chronic maintenance hemodialysis [25]. Cuisset et al. also found that no significant difference between patients with or without CKD by two platelet function tests in ACS population [26]. Furthermore, compared with non-CKD patients, CKD patients were found to have lower response to aspirin therapy but the platelet response to dual-antiplatelet therapy was not different between CKD and non-CKD patients [27]. Our data showed clopidogrel is beneficial not only in the non-CKD population but also in the CKD subjects.

### Clopidogrel and Cardiovascular Outcome in CKD Population

In the CURE trial clopidogrel was beneficial and safe in patients with and without chronic kidney disease [28]. However, clopidogrel in mild or moderate CKD patients may not have the same beneficial effect as it does in patients with normal renal function in the CREDO trial [29]. Furthermore, retrospective study found a low response to clopidogrel might be a risk factor for the poorer outcomes in patients with stage 3 to 5 CKD compared with patients with better renal function [30]. Meta-analysis had also shown little or no effect on all-cause or cardiovascular mortality or on myocardial infarction after add-on clopidogrel in patients with CKD [31]. Because of conflict data, more studies are needed to clarify the effect of clopidogrel on the cardiovascular disease in the CKD population. Although we had no bench data to show the antiplatelet effect of clopidogrel in the CKD population, CKD patients receiving clopidogrel treatment also have lower cardiovascular events as in the non-CKD population in the real-world study.

### In-hospital Bleeding and ACS Outcome

In-hospital bleeding (IHB) is associated with short-, intermediate-, and long-term mortality among patients hospitalized for ACS [3], [4], [32]. Patients with IHB after primary PCI in ST segment elevation ACS have significantly increased 3-year rates of morbidity and mortality [33]. The deleterious effect of major bleeding was observed within 1 month, between 1 month and 1 year, and between 1 and 3 years. In patients with non-ST segemnt elevation ACS cumulative mortality was also higher in those who had bleeding vs. those without bleeding at 30 days, 1 year, and 3 years [32]. In our study patients receiving clopidogrel had no increase the risk of in-hospital TIMI bleeding in the whole, CKD and non-CKD population even after adjusting the related variables. Our data could support to safely use clopidogrel in the CKD population in the ACS.

### Limitations

This study has three main limitations. Firstly, it is a nonrandomized, observational and post-hoc analysis study. Furthermore, there were few patients not treated with clopidogrel in this study. Nonetheless, this study provides valuable real-world data on the current practices across the full spectrum of ACS in a CKD endemic area, which could help to improve the ACS management in the CKD population. Second, we did not evalaute the antiplatelet effects of clopidogrel, which might provide the data for individualized therapy in the CKD population. Third, the renal endpoint and bleeding events were not routinely collected after discharge in this registry. Whether those with clopidogrel had better renal outcome is unknown.

### Conclusion

In this real-word registry we found patients receiving clopidogrel treatment had lower risk of death and cardiovascular events at 12 months not only in the non-CKD but also in the CKD population. Patients with CKD and no clopidogrel treatment had the worst outcome. Furthermore, they had additively detrimental effect on the cardiovascular outcome. Thus, clopidogrel use and preventing CKD in ACS patients are important for the eventual cardiovascular risk reduction.
